# Association Between Lateral Placenta and Adverse Maternal and Perinatal Outcomes: A Systematic Review and Meta‐Analysis

**DOI:** 10.1111/1471-0528.70278

**Published:** 2026-06-07

**Authors:** Joseph Arkorful, Juliana Nyamson, Theophilus Adu‐Bredu, Kwame Adu‐Bonsaffoh, Joyce Browne, Albert D. Piersson, Kitty Bloemenkamp

**Affiliations:** ^1^ Department of Obstetrics, Division Woman and Baby Wilhelmina Children Hospital, University Medical Center, Utrecht University Utrecht the Netherlands; ^2^ Department of Imaging Technology & Sonography University of Cape Coast Cape Coast Ghana; ^3^ Nuffield Center of Women's Health, University of Oxford Oxford UK; ^4^ Department of Obstetrics and Gynaecology University of Ghana, School of Medical Sciences Accra Ghana; ^5^ Julius Global Health, Julius Center for Health Sciences and Primary Care, University Medical Center Utrecht Utrecht the Netherlands; ^6^ Department of Diagnostic Radiography & Medical Imaging York St John University York UK

**Keywords:** adverse maternal outcomes, adverse perinatal outcomes, lateral placenta, lateral placentation, meta‐analysis, placental dysfunction, systematic review

## Abstract

**Background:**

Adverse maternal and perinatal outcomes such as preeclampsia, small‐for‐gestational age (SGA) and preterm birth remain major global health concerns. Beyond known high‐risk placental features, emerging evidence suggests lateral placenta to be associated with impaired uteroplacental blood flow resulting in placenta dysfunction and adverse outcomes. A better understanding of these associations requires synthesizing both crude and adjusted effect estimates from available evidence.

**Objectives:**

To comprehensively review and synthesize available evidence on the association between lateral placenta and adverse maternal and perinatal outcomes.

**Search Strategy:**

MEDLINE (PubMed), EMBASE, Scopus and Cochrane CENTRAL were searched on 25th August, 2025.

**Selection Criteria:**

Studies that assessed the association between lateral placentation and adverse maternal and perinatal outcomes in singleton pregnancies.

**Data Collection and Analysis:**

Data were independently extracted by two reviewers. The random‐effects model was used to pool estimates of both crude and adjusted odds ratios (ORs) with corresponding 95% confidence interval (CI). Statistical heterogeneity was assessed by the *I* ^2^ statistic and Cochran's *Q* test.

**Main Results:**

Twenty one eligible studies with a total of 162 727 singleton pregnancies were included in the meta‐analyses. Lateral placenta was associated with preeclampsia (OR = 1.65, 95% CI: 1.25, 2.19, *I* ^2^ = 41.0%), SGA (OR = 1.40, 95% CI: 1.17, 1.68, *I* ^2^ = 69.0%), preterm birth < 34 weeks (OR = 2.10, 95% CI: 1.62, 2.72, *I* ^2^ = 0.0%), preterm birth < 37 weeks (OR = 1.50, 95% CI: 1.26, 1.80, *I* ^2^ = 60.5%), retained placenta (OR = 2.52, 95% CI: 1.60, 3.95, *I* ^2^ = 87.7%), and non‐vertex foetal presentation at birth (OR = 1.50, 95% CI: 1.19, 1.89, *I* ^2^ = 28.6%). Two individual studies reported independent association between lateral placenta and preeclampsia; with adjusted odds ratio (aOR) of 2.04 (95% CI: 1.28, 3.25) and 1.32 (95% CI: 1.04, 1.67). Pooled adjusted OR (95% CI) demonstrated increased odds of SGA (aOR = 1.84, 95% CI: 1.33, 2.53, *I* ^2^ = 0.0%), and retained placenta (aOR = 4.43, 95% CI: 1.70, 11.53, *I* ^2^ = 76.1%). Marginal increase in odds was noted for preterm birth < 34 weeks (aOR = 2.14, 95% CI: 1.34, 3.41, *I* ^2^ = 0.00%) and preterm birth < 37 weeks (aOR = 1.54, 95% CI: 1.11, 2.13, *I* ^2^ = 38.8%).

**Conclusions:**

Lateral placenta is associated with increased odds of preeclampsia, SGA, preterm birth, non‐vertex foetal presentation, and retained placenta. After controlling for confounders, lateral placenta remained independently associated with increased odds of SGA, preterm birth, and retained placenta. More studies that adjust for confounders are, however, needed to further clarify and strengthen the evidence base of this independent association.

## 1 Introduction

The placental location, defined by its attachment site within the uterine cavity, is routinely assessed during mid‐trimester obstetric ultrasound scans. While anterior, posterior, fundal or lateral placenta are generally regarded as physiological variants, emerging evidence suggests that placental location is associated with outcomes such as preeclampsia, antepartum haemorrhage, small‐for‐gestational age (SGA), preterm birth, retained placenta and stillbirth [1, 2, 3, 4, 5]. Although the biological and physiological linkage of placental location and adverse pregnancy outcomes is not well elucidated, it is believed that the contours of different regions of the uterine cavity have an effect on placentation and uteroplacental blood perfusion. For instance, it is argued that due to the relatively sharp angles of the lateral aspects of the uterine cavity, in contrast to the flattened surface of the central aspect (anterior or posterior), there is the possibility of suboptimal placental attachment and possibly placental dysfunction. Furthermore, for lateral placenta, the side of placental attachment (either right or left) is thought to receive blood supply primarily from the single ipsilateral uterine artery, unlike central placentation, which benefits from a dual supply from both uterine arteries. This uneven perfusion may impair uteroplacental blood flow, potentially resulting in uteroplacental insufficiency and increased risk of adverse pregnancy outcomes [6, 7].

Despite the likely association between lateral placenta and adverse pregnancy outcomes, evidence from primary studies remains inconsistent. For instance, while several primary studies have reported associations between lateral placenta and outcomes such as preeclampsia and SGA [1, 4, 8, 9, 10, 11], other studies [12, 13, 14, 15] have found no such association. This inconsistency in the literature highlights the need for a comprehensive synthesis of the available evidence to understand the potential role of lateral placenta as an additional marker for identifying pregnancies at higher risk of poor outcomes.

Previous meta‐analyses [16, 17, 18] investigating the association between lateral placenta and adverse pregnancy outcomes have relied solely on crude odds ratios to interpret their findings. This represents a significant gap in the current evidence, as crude estimates do not account for the influence of potential confounders and therefore cannot fully capture the true nature of the association between lateral placenta and adverse outcomes. To better elucidate the independent association between lateral placenta and adverse maternal and perinatal outcomes, it is essential to synthesize evidence from studies that report adjusted effect estimates, after controlling for confounders such as maternal age, parity, ethnicity, comorbidities, and other obstetric factors.

This current systematic review and meta‐analysis therefore aimed to update the current evidence by pooling both crude and multivariable‐adjusted effect estimates, in order to determine whether lateral placenta is independently associated with adverse maternal and perinatal outcomes. This ensures a more robust and clinically meaningful estimate of risk, improves understanding of the prognostic value of lateral placenta for adverse pregnancy outcomes, and informs future research and clinical decision‐making.

## 2 Methods

### 2.1 Protocol and Registration

This systematic review and meta‐analysis was reported according to the Preferred Item for Systematic Review and Meta‐Analysis statement [19]. The review protocol was registered with PROSPERO (registration number: CRD42024528787).

### 2.2 Eligibility Criteria

#### 2.2.1 Study Design

Prospective or retrospective cohort studies and case–control studies conducted in either a high‐income country (HIC) or low‐ and middle‐income country (LMIC) that investigated the association between ultrasound‐determined placental location and adverse maternal and perinatal outcomes in singleton pregnancies. Case reports, case series, reviews, editorials, conference proceedings, and articles with missing full text were excluded.

#### 2.2.2 Participants

Pregnant women with a single non‐anomalous foetus. Multiple gestation was excluded. Also, women with low‐lying placenta, placenta previa and placenta accreta spectrum were excluded.

#### 2.2.3 Determinant

Ultrasound‐determined placental locations of interest were those reported as not low‐lying, defined according to International Society of Ultrasound in Obstetrics and Gynaecology (ISUOG) guidelines as placenta with a leading edge more than 20 mm away from the internal cervical OS [20]. Studies were therefore included if they investigated non low‐lying placentae in the second or third trimester, and further categorized placental location as: lateral, defined as placenta predominantly occupying either the right or left side walls of the uterine cavity (i.e., placenta occupying three‐quarters of either right or left lateral uterine walls, or if more than two‐thirds of the placenta were right or left to the midline); anterior, defined as placenta occupying the front wall of the uterine cavity; posterior, defined as placenta occupying the posterior wall of the uterine cavity; and fundal, defined as placenta occupying the fundal aspect of the uterine cavity [2, 4]. Low‐lying placenta, placenta previa and placenta accreta spectrum were therefore excluded. Furthermore, articles with ambiguous placental location definition and categorization were excluded. Also, studies that assessed placental location after delivery, such as through manual exploration, were excluded.

#### 2.2.4 Outcomes

The primary outcomes were as follows:

1. Small‐for‐gestational age (SGA), defined as a new‐born birthweight below the 10th percentile for their gestational age [21].
2. Preterm birth, defined as the birth of a live baby before the 37th week of pregnancy.
3. Stillbirth, defined according to the WHO as death of the foetus (at ≥ 28 weeks gestational age) before or during childbirth [22].
4. Low birth weight according to WHO definition as weight at birth < 2500 g [23].
5. Low APGAR score < 7 at the 5th minute [24].
6. Preeclampsia, as defined by the individual primary studies
7. Other neonatal adverse outcomes such as NICU admission, hypoxic ischaemic events, respiratory distress syndrome, seizures and sepsis.

Secondary outcomes were caesarean section (c‐section) due to non‐reassuring foetal status, non‐vertex foetal presentation at birth, ante‐partum haemorrhage, post‐partum haemorrhage (as defined by the individual primary studies), and retained placenta requiring manual removal.

### 2.3 Data Sources and Search Strategy

A literature search was conducted in MEDLINE (PubMed), EMBASE, Scopus and Cochrane Library (CENTRAL) on 25th August, 2025, for available studies from inception to the date of search. A librarian from Utrecht University assisted in developing the search strategy and search blocks used for the literature search (Appendix S1).

No restrictions on language, geographical setting or publication date were applied during the search. Articles published in languages other than the English language were screened by title and abstract using available English translation software. Further translation of full‐text articles for potentially eligible studies was undertaken using translation software to determine eligibility and extract data.

Reference lists of the eligible articles were screened to identify additional articles (snowballing).

### 2.4 Study Selection

The retrieved articles from the database search were exported to Endnote 21 for removal of duplicate records. Afterwards, the remaining non‐duplicated records were transferred to Rayyan [25] for screening and selection of eligible articles. Two reviewers (JA and JN) independently screened the titles and abstracts for identification of eligible studies. Full texts of the eligible studies were then retrieved and screened by the same two reviewers (J.A. and J.N.). In cases of any disagreements, consensus was reached by the same two reviewers.

### 2.5 Data Extraction

Data extraction was carried out by two reviewers (J.A. and J.N.) using a pre‐piloted data extraction sheet agreed upon by the research team. Extracted data included information on authors and publication year, study country of origin, sample size, study design, gestational age at the time of recruitment, placental location explored, and outcomes investigated.

#### 2.5.1 Placental Location Categorization

Based on commonly reported ultrasound placental location and categorization approaches described in the literature [2, 4, 11], placental location was extracted from the eligible articles and categorized as lateral when the placenta was predominantly located on the right or left uterine wall and central when the placenta was located at the anterior, posterior, or fundal aspect of the uterine wall.

In situations where any missing data was encountered, corresponding authors of these articles were contacted once by email.

### 2.6 Risk of Bias Assessment

Study quality and risk of bias (RoB) assessment of the included studies was conducted using the Newcastle–Ottawa scale (NOS) [26]. For cohort studies, the NOS utilizes a checklist for assessing study quality under three broad domains: namely selection of cohorts, comparability of cohort, and assessment of outcome. For case–control studies, the three domains used are selection of cases and controls, comparability of cases and controls, and ascertainment of exposure. A score of good, fair, or poor quality is assigned to each study, based on performance under each domain. J.A. and J.N. independently performed the RoB assessment. Any disagreements were resolved by consensus.

### 2.7 Patient and Public Involvement

No patient or public was involved in the design of this study.

### 2.8 Statistical Analysis

We extracted primary 2 × 2 contingency data from all eligible studies to calculate study‐specific and pooled estimates of unadjusted odds ratios (ORs) with corresponding 95% confidence intervals (CI). For studies that reported only effect estimates (unadjusted ORs with 95% CIs), we back‐calculated the corresponding 2 × 2 data from reported event rates and sample sizes.

Given the binary nature of the maternal and perinatal outcomes and the expected heterogeneity across studies, we performed meta‐analysis using the Mantel–Haenszel (MH) method within a random‐effects framework, with between‐study variance estimated using the DerSimonian–Laird (DL) approach. This model is preferred since it accounts for both within‐study and between‐study variability, providing a more conservative estimate of the overall effect. Furthermore, the MH method was considered appropriate, given that some of the included studies had small sample sizes and zero events in one arm.

For secondary analysis of studies reporting adjusted ORs, we extracted the estimates and their corresponding 95% CIs as reported by the individual studies, log‐transformed them, and then applied a random‐effects model to calculate the pooled adjusted OR (95% CI), following methods outlined in the Cochrane Handbook [27]. This analysis was performed to assess whether adjustment for confounders altered the estimated association between lateral placenta and adverse maternal and perinatal outcomes.

Statistical heterogeneity was assessed using the *I* ^2^ statistic and Cochran's *Q* test. In interpreting *I* ^2^ statistic, approximately ≤ 25% connoted low heterogeneity, 25% –75% connoted moderate heterogeneity, and > 75% connoted considerable heterogeneity [28]. In the event where the detection of heterogeneity was not reliable due to small sample size, data were pooled using a random effect model.

Where at least ten studies were available for an outcome, potential publication bias was assessed through visual inspection of funnel plots and quantitatively with Egger's regression test.

Also, where substantial heterogeneity (*I* ^2^ > 50%) was identified for an outcome, potential sources were explored through subgroup analyses based on study design (cohort vs. case–control) where feasible.

Furthermore, in examining the consistency and robustness of our findings, a sensitivity analysis was conducted using the leave‐one‐out method.

All statistical analysis was conducted using the R software packages ‘meta’ and ‘metafor’. All *p*‐values were two‐sided at the 5% significant level.

## 3 Results

### 3.1 Study Selection

Through the database search, a total number of 13 117 records were identified. After duplicate records were removed, 8264 records remained for title and abstract screening. After screening, 29 records remained for full‐text review. Eight articles were excluded at full‐text review, with 21 articles finally remaining for inclusion (Figure 1). The list of excluded full‐text articles with reasons for exclusion is provided in Table S2.

**FIGURE 1:**
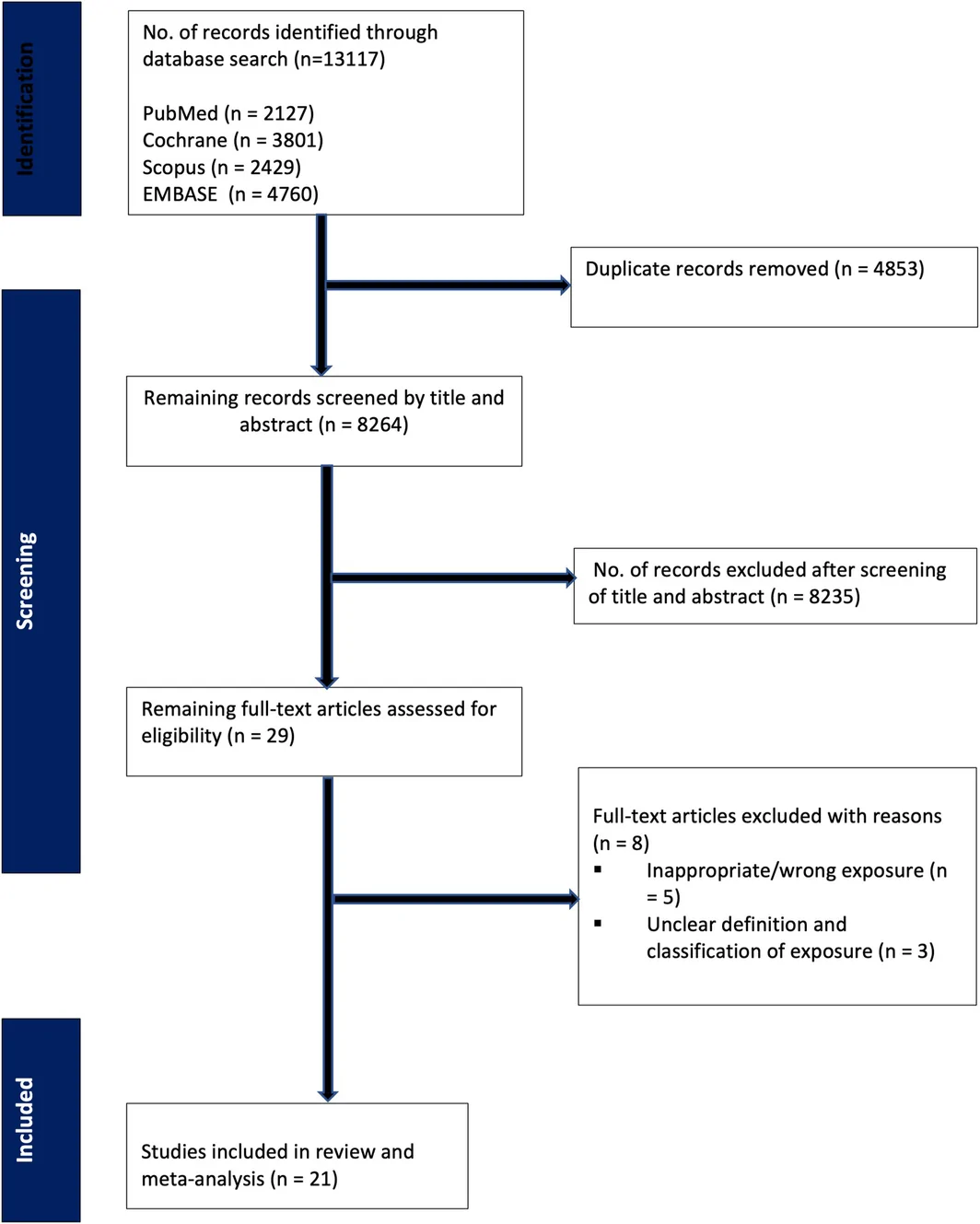
PRISMA flow diagram.

### 3.2 Study Characteristics

The 21 eligible studies included a total of 162 727 singleton pregnancies, with the studies originating from USA (*n* = 2), Canada (*n* = 1), Greece (*n* = 2), Sweden (*n* = 2), Israel (*n* = 4), Italy (*n* = 3), China (*n* = 1), Turkey (*n* = 1), India (*n* = 2), Iran (*n* = 1) and Nigeria (*n* = 1). One multicenter study was conducted across both the USA and Australia. The majority of the studies (*n* = 18) were population‐based cohort studies, while the rest (*n* = 3) were case–control studies (Table S1).

### 3.3 Quality Assessment of Risk of Bias Within Studies

Of the 21 included studies, eight [1, 2, 4, 5, 9, 11, 13, 15] were assessed as having a low risk of bias, while the remaining thirteen [3, 8, 10, 12, 14, 29, 30, 31, 32, 33, 34, 35, 36] were rated as having a moderate risk of bias. No study was rated as having a high risk of bias. The moderate risk of bias rating was primarily attributed to a lack of adjustment for important confounders under the ‘comparability of cohort’ domain of NOS (Table S3).

### 3.4 Association Between Lateral Placenta and Adverse Maternal and Perinatal Outcomes

#### 3.4.1 Preeclampsia

The analysis demonstrated that pregnant women with lateral placenta had increased odds of developing preeclampsia, as compared to women with central placenta (OR = 1.65, 95% CI: 1.25, 2.19, *I* ^2^ = 41.0%, 12 studies) (Table 1, Figure 2a).

**TABLE 1:** Pooled odds ratios (ORs) of maternal and perinatal outcomes associated with lateral placenta.

Outcome	Studies (*n* ^ref^)	Cases (*n*/*N*)	Pooled OR (95% CI)^a^	*I* ^2^ (%)
Maternal outcomes				
Preeclampsia	12 [1, 2, 3, 4, 8, 10, 11, 12, 14, 32, 33, 34]	3590/111469	1.65 (1.25–2.19)	41.0
Postpartum haemorrhage	5 [1, 4, 8, 10, 13]	5486/94405	1.01 (0.63–1.64)	79.6
Caesarean section due to non‐reassuring foetal status	3 [4, 10, 12]	935/16810	1.18 (0.84–1.64)	31.9
Retained placenta requiring manual removal	6 [1, 4, 5, 15, 35, 36]	4170/134503	2.52 (1.60–3.95)	87.7
Perinatal outcomes				
Small‐for‐gestational age (SGA)	12 [1, 2, 4, 8, 9, 10, 11, 13, 15, 30, 31, 32]	8555/138681	1.40 (1.17–1.68)	69.0
Preterm birth < 34 weeks	4 [1, 2, 4, 32]	923/96423	2.10 (1.62–2.72)	0.0
Preterm birth < 37 weeks	10 [1, 2, 3, 4, 10, 11, 13, 15, 29, 32]	7939/138037	1.50 (1.26–1.80)	60.5
5th minute Apgar score < 7	6 [1, 2, 4, 10, 13, 15]	992/119019	1.29 (0.85–1.96)	40.8
Non‐vertex foetal presentation at birth	4 [1, 4, 13, 29]	3621/94012	1.50 (1.19–1.89)	28.6
Neonatal intensive care unit (NICU) admission	5 [2, 4, 8, 10, 15]	5593/42087	1.23 (0.97–1.57)	36.9
Stillbirth	3 [4, 10, 13]	54/19921	1.07 (0.33–3.49)	0.0

^a^ Pooled odds ratio (OR) by random effects model.

**FIGURE 2:**
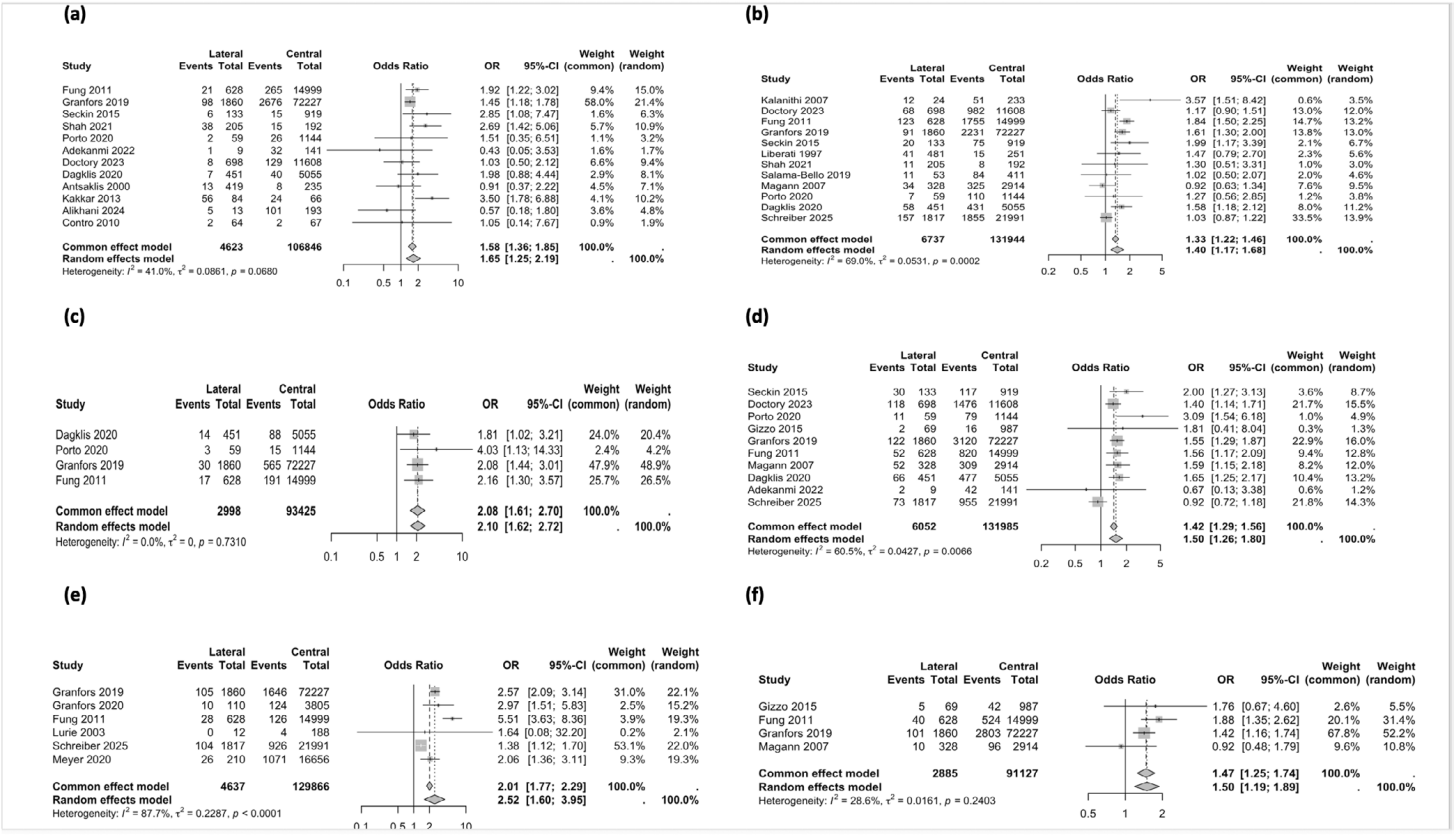
Meta‐analysis of the association between lateral placenta and (a) pre‐eclampsia, (b) small‐for‐gestational age, (c) preterm birth < 34 weeks, (d) preterm birth < 37 weeks, (e) retained placenta, (f) non‐vertex foetal presentation.

Assessment of potential publication bias by visual inspection of the funnel plot showed a symmetrical distribution of studies around the pooled effect size, with no clustering or asymmetry, suggesting a low likelihood of publication bias (Figure S1). Furthermore, the Egger's test for funnel plot asymmetry confirmed the absence of significant publication bias (*p* = 0.93), and no meaningful small‐study effects (estimated bias coefficient = 0.06, SE = 0.64).

#### 3.4.2 Small‐For‐Gestational‐Age (SGA)

The analysis demonstrated that pregnant women with lateral placenta had increased odds of giving birth to SGA babies, as compared to women with central placenta (OR = 1.40, 95% CI: 1.17, 1.68, *I* ^2^ = 69.0%, 12 studies) (Table 1, Figure 2b).

Visual inspection of the funnel plot showed a symmetrical distribution of studies around the pooled effect size, with no clustering or asymmetry, suggesting a low likelihood of publication bias (Figure S2). This was further supported by Egger's test (*p* = 0.59), which indicated no significant small‐study effects detected (estimated bias coefficient = 0.60, SE = 1.07).

To explore the sources of heterogeneity, we performed a subgroup analysis by study design (cohort vs. case–control). The case–control study demonstrated higher estimated odds of SGA (OR = 3.57, 95% CI: 1.51, 8.42), compared with the cohort studies (OR = 1.36, 95% CI: 1.14, 1.62, *I* ^2^ = 67.5%). Test for subgroup differences indicated significant between‐group heterogeneity (*Q* = 4.73, *p* = 0.03) (Figure S4).

#### 3.4.3 Preterm Birth < 34 Weeks

The analysis demonstrated that a lateral placenta was associated with increased odds of preterm birth (< 34 weeks) compared with central placenta (OR = 2.10, 95% CI: 1.62, 2.72, *I* ^2^ = 0.0%, 4 studies) (Table 1, Figure 2c).

#### 3.4.4 Preterm Birth < 37 Weeks

The analysis demonstrated that a lateral placenta was associated with increased odds of preterm birth (< 37 weeks) compared with central placenta (OR = 1.50, 95% CI: 1.26, 1.80, *I* ^2^ = 60.5%, 10 studies) (Table 1, Figure 2d).

Assessment by visual inspection of the funnel plot showed a symmetrical distribution of studies around the pooled effect size, with no clustering or asymmetry, suggesting a low likelihood of publication bias (Figure S3). This was supported by Egger's test (*p* = 0.53), which indicated no significant small‐study effects detected (estimated bias coefficient = 0.71, SE = 1.09).

#### 3.4.5 Retained Placenta Requiring Manual Removal

The analysis demonstrated that pregnancies with laterally located placentae were associated with significantly increased odds of retained placenta requiring manual removal at delivery compared with those with centrally located placentae (OR = 2.52, 95% CI: 1.60, 3.95, *I* ^2^ = 87.7%, 6 studies) (Table 1, Figure 2e).

#### 3.4.6 Non‐Vertex Foetal Presentation at Birth

The analysis showed that pregnancies with laterally located placentae were associated with increased odds of non‐vertex foetal presentation at birth compared with those with centrally located placentae (OR = 1.50, 95% CI: 1.19, 1.89, *I* ^2^ = 28.6%, 4 studies) (Table 1, Figure 2f).

#### 3.4.7 Other Maternal and Perinatal Outcomes

The analyses did not demonstrate any statistically significant association between lateral placenta and caesarean section due to non‐reassuring foetal status (OR = 1.18, 95% CI: 0.84, 1.64, *I* ^2^ = 31.9%, 3 studies) (Table 1, Figure S5), post‐partum haemorrhage (OR = 1.01, 95% CI: 0.63, 1.64, *I* ^2^ = 79.6%, 5 studies) (Table 1, Figure S6), 5‐min Apgar score < 7 (OR = 1.29, 95% CI: 0.85, 1.96, *I* ^2^ = 40.8%, 6 studies) (Table 1, Figure S7), NICU admission (OR = 1.23, 95% CI: 0.97, 1.57, *I* ^2^ = 36.9%, 5 studies) (Table 1, Figure S8), and stillbirth (OR = 1.07, 95% CI: 0.33, 3.49, *I* ^2^ = 0.0%, 3 studies) (Table 1, Figure S9).

#### 3.4.8 Sensitivity Analyses

For preeclampsia, the pooled ORs after sequentially omitting each study ranged from 1.50 (95% CI: 1.28–1.76) to 1.77 (95% CI: 1.40–2.25), with all 95% CIs remaining statistically significant (*p* < 0.0001) (Figure S10).

For SGA, the pooled ORs after sequential omitting each study ranged from 1.25 (95% CI: 1.13–1.38) to 1.49 (95% CI: 1.34–1.65), with all 95% CIs remaining statistically significant (*p* < 0.0001) (Figure S11).

For preterm birth < 34 weeks, the pooled ORs after sequential omitting each study ranged from 2.03 (95% CI: 1.56–2.65) to 2.17 (95% CI: 1.62–2.90), with all 95% CIs remaining statistically significant (*p* < 0.0001) (Figure S12).

For preterm birth < 37 weeks, the pooled ORs after sequential omitting each study ranged from 1.38 (95% CI: 1.24–1.54) to 1.56 (95% CI: 1.41–1.73), with all 95% CIs remaining statistically significant (*p* < 0.0001) (Figure S13).

For retained placenta, the pooled ORs after sequential omitting each study ranged from 1.76 (95% CI: 1.49–2.08) to 2.72 (95% CI: 2.32–3.20), with all 95% CIs remaining statistically significant (*p* < 0.0001) (Figure S14).

For non‐vertex foetal presentation, the pooled ORs after sequential omitting each study ranged from 1.37 (95% CI: 1.13–1.66) to 1.59 (95% CI: 1.19–2.10), with all 95% CIs remaining statistically significant (*p* < 0.001) (Figure S15).

The analyses therefore indicate that the overall results for each outcome are robust, with no single study unduly influencing the results.

### 3.5 Secondary Analysis: Adjustment for Confounders

The analysis of studies that adjusted for potential confounders (Table S4) demonstrated that lateral placenta was significantly associated with increased odds of SGA (OR = 1.84, 95% CI: 1.33, 2.53, *I* ^2^ = 0.0%, 3 studies) (Table 2, Figure S16), preterm birth < 34 weeks (OR = 2.14, 95% CI: 1.34, 3.41, *I* ^2^ = 0.0%, 3 studies) (Table 2, Figure S17), preterm birth < 37 weeks (OR = 1.54, 95% CI: 1.11, 2.13, *I* ^2^ = 38.8%, 4 studies) (Table 2, Figure S18), and retained placenta requiring manual removal (OR = 4.43, 95% CI: 1.70, 11.53, *I* ^2^ = 76.1%, 3 studies) (Table 2, Figure S19).

**TABLE 2:** Pooled adjusted odds ratios (aORs) of maternal and perinatal outcomes associated with lateral placenta.

Outcome	Studies (*n* ^ref^)	Pooled aOR (95% CI)^a^	*I* ^2^ (%)
Maternal outcomes			
Preeclampsia	2 [1, 4]	1.56 (0.11–23.20)	62.5
Retained placenta requiring manual removal	3 [1, 4, 5]	4.43 (1.70–11.53)	76.1
Perinatal outcomes			
Small‐for‐gestational age (SGA)	3 [1, 4, 9]	1.84 (1.33–2.53)	0.0
Preterm birth < 34 weeks	3 [1, 2, 4]	2.14 (1.34–3.41)	0.0
Preterm birth < 37 weeks	4 [1, 2, 4, 11]	1.54 (1.11–2.13)	38.8

^a^ Pooled adjusted odds ratio (OR) by random effects model.

However, the pooled analysis for preeclampsia showed no statistically significant association (OR = 1.56, 95% CI: 0.11, 23.20, *I* ^2^ = 62.5%, 2 studies) (Table 2, Figure S20).

## 4 Discussion

### 4.1 Main Findings

This comprehensive systematic review and meta‐analysis synthesized evidence from 21 observational studies that recruited a total of 162 727 singleton pregnancies to investigate the association between lateral placenta and adverse maternal and perinatal outcomes. The overall findings indicate that lateral placenta is significantly associated with increased odds of preeclampsia, small‐for‐gestational‐age (SGA), preterm birth, non‐vertex foetal presentation, and retained placenta requiring manual removal. Of note, these associations were seen to be consistent after sensitivity analyses, confirming the robustness of the findings, irrespective of study design or study context.

In secondary meta‐analyses, pooled estimates from studies reporting adjusted OR (95% CI) after controlling for possible confounders demonstrated further increased odds of SGA and retained placenta. For preterm birth < 34 weeks and < 37 weeks, only a marginal increase in odds was observed.

### 4.2 Strengths and Limitations of Study

A key strength of this meta‐analysis is the inclusion of a large cohort of 162 727 singleton pregnancies from studies conducted across North America, Europe, Asia, Africa and Oceania. This approach enhances statistical power, improves generalizability of findings, and offers a global perspective on the association between lateral placenta and adverse maternal and perinatal outcomes.

Previous meta‐analysis that investigated the relationship between placental location and adverse pregnancy outcomes [16, 17, 18] have primarily relied on pooled crude odds ratios to draw inferences. To the best of our knowledge, this is the first meta‐analysis to pool both crude and adjusted odds ratios for adverse maternal and perinatal outcomes associated with lateral placenta. This approach made it possible to better assess whether lateral placenta was independently associated with these outcomes, while accounting for confounding factors that might obscure the true association.

Another strength lies in the rigorous methodological approach, including sensitivity analyses which assessed the robustness and validity of the primary results. Furthermore, the absence of publication bias, as evidenced by funnel plots and Egger's regression tests, supports the reliability of our findings.

Despite the strengths of this study, some limitations should be acknowledged. First, although all included studies were rated as having low or moderate risk of bias, none were randomized controlled trials, since such trials are not currently available in the literature. Therefore, the possibility of residual confounding cannot be excluded. Second, some of the studies were retrospective, relying on existing ultrasound reports from medical records instead of standardized, pre‐specified assessments. This may have introduced misclassification bias, since differences in reporting quality or how placental location was categorized could affect accuracy. Additionally, within the current literature, only a few studies have adjusted for confounders while assessing the independent association between lateral placenta and adverse maternal and perinatal outcomes, representing a limitation. Furthermore, despite some agreement in adjustment of key variables such as maternal age and maternal background characteristics, differences in additional confounder adjustment were observed. This variability, mainly due to differences in study design, warrants some caution in the interpretation of the results.

### 4.3 Interpretation of Findings

This meta‐analysis was conducted on the premise that, compared to central placenta, lateral placenta could influence adverse pregnancy outcomes as a result of suboptimal placental attachment to the uterine wall and impaired uteroplacental perfusion [6]. This hypothesis is supported by evidence depicting increased resistance to blood flow, as assessed by uterine artery Doppler, in pregnancies with lateral placentas [7, 30].

Our findings indicate that lateral placenta is associated with increased odds of adverse outcomes such as preeclampsia, retained placenta, SGA, preterm birth and non‐vertex foetal presentation. These findings are consistent with a previous study that discovered an association between lateral placenta and SGA, preeclampsia and preterm birth [16]. Similar to a previous study [17], we found no statistically significant association between lateral placenta and 5‐min Apgar score < 7, NICU admission and stillbirth. However, unlike a previous study [18] that found no association with preterm birth, our study demonstrated increased odds for both preterm birth < 34 and < 37 weeks.

From our secondary analysis, the increased odds of SGA after controlling for advanced maternal age, parity, ethnicity and chronic hypertension is noteworthy. This observation suggests a likely biological plausibility of an association between lateral placenta and impaired uteroplacental perfusion, a recognized underlying cause of SGA. However, despite all three studies [1, 4, 9] adjusting for key baseline confounders such as maternal age, ethnicity and chronic hypertension (Table S4), we noticed additional adjustments of confounders that were not consistent across the studies. For instance, Fung et al. [4] additionally adjusted for nulliparity and body mass index, which were not accounted for in the other two studies. Similarly, Granfors et al. [1] additionally adjusted for smoking in early pregnancy, infant sex, and in vitro fertilization. Due to these variations in confounder adjustment, the pooled adjusted estimates should therefore be interpreted with caution.

In this review, the two individual studies by Fung et al. [4] and Granfors et al. [1] reported significant independent associations between lateral placenta and preeclampsia, with adjusted odds ratios (aOR) of 2.04 (95% CI: 1.28, 3.25) and 1.32 (95% CI: 1.04, 1.67), respectively (Table S4). However, the meta‐analysis of these two studies yielded a pooled aOR of 1.56 with a wide confidence interval (95% CI: 0.11, 23.20) (Table 2) that failed to reach statistical significance. This result is likely driven by the very small number of studies (*n* = 2), which influenced the meta‐analytic model by limiting its stability, resulting in imprecision of the pooled estimate, as reflected in the wide confidence interval. Furthermore, with few studies, estimation of between‐study heterogeneity is often unreliable, which may have further contributed to the uncertainty of the pooled effect. Nevertheless, the consistent direction of effect across the individual studies and pooled estimate suggests a potential independent association between lateral placenta and preeclampsia.

Unlike previous meta‐analyses, our study is the first to separately report on the association between lateral placenta and preterm birth < 34 weeks and preterm birth < 37 weeks, recognizing the clinical significance of these outcomes for both short‐term and long‐term neonatal well‐being. We found that women with lateral placenta had higher odds of preterm birth < 34 weeks compared with preterm birth < 37 weeks (OR = 2.10 vs. 1.50). This is supported by the secondary analysis, where the pooled adjusted ORs remained consistent, with no dampening effect after controlling for confounders such as maternal age, parity and antepartum haemorrhage. These findings further support the notion that a lateral placenta may be a risk factor for impaired uteroplacental perfusion and subsequent placental dysfunction, thereby associated with preeclampsia and SGA; conditions that can often necessitate early delivery to prevent serious complications.

The increased odds of non‐vertex foetal presentation in pregnancies with lateral placenta may be explained by placental attachment to the lateral uterine wall, which may restrict the foetus's movement from a non‐vertex to a vertex position. This finding is consistent with previous studies that have identified lateral and fundal placental location as risk factors for non‐vertex presentation [37, 38].

The increased odds of retained placenta with lateral placenta can be explained by impaired placentation and hypoperfusion. These mechanisms are established contributors to oxidative stress and the development of preeclampsia, preterm birth, and SGA [39]. It is therefore plausible that pregnancies with laterally located placentae complicated by these outcomes are also at increased risk of retained placenta. Supporting this, a histological study [40] demonstrated features of hypoperfusion in retained placentae specimens, similar to those observed in preeclamptic placentae specimens.

### 4.4 Clinical and Research Implications

Given the global burden of preeclampsia, SGA, preterm birth, and postpartum haemorrhage secondary to retained placenta, particularly in LMICs, this review offers valuable insights into the role of routine ultrasound‐determined placental location in identifying pregnancies at increased risk. Placental location, often regarded as a normal anatomical variant, has historically received little attention in predicting adverse pregnancy outcomes. To date, no obstetric guideline recommends the use of lateral placenta as a routine mid‐trimester risk indicator, primarily due to inconsistencies in the existing evidence.

However, from our findings, identifying lateral placenta during routine mid‐trimester ultrasound scan could potentially serve as an additional prenatal risk marker.

Although third‐trimester ultrasound is well established to be beneficial in high‐risk pregnancies, some studies [41, 42] do not support its routine use in unselected low‐risk populations. The major clinical dilemma lies in risk stratifying within the general population to identify women who may require additional maternal and foetal surveillance. In this context, the presence of lateral placenta could be considered as part of second‐trimester risk stratification, with pregnancies flagged as high‐risk offered additional surveillance during the third trimester.

Insights from our secondary meta‐analysis of studies that reported adjusted odds ratios suggest a likely independent association between lateral placenta and adverse maternal and perinatal outcomes. However, given the limited number of studies that reported adjusted ORs, as well as variability in the confounders adjusted, these findings should be interpreted with caution. More well‐designed prospective studies with consistent and adequate adjustment of confounders are needed to better appreciate these associations. In particular, when determining the independent association between lateral placenta and preeclampsia, these studies should account for aspirin use in high‐risk pregnancies, either by adjusting for it as a covariate or by conducting subgroup analyses stratified by aspirin use or not. Again, for non‐vertex foetal presentation, investigators should either adjust for uterine anomalies or clearly state the exclusion of women with such anomalies, as these represent an important potential confounder for this outcome.

## 5 Conclusion

Our findings demonstrate lateral placenta to be associated with increased odds of preeclampsia, small‐for‐gestational age, preterm birth, non‐vertex foetal presentation, and retained placenta. After controlling for confounders, lateral placenta remains independently associated with increased odds for SGA, preterm delivery, and retained placenta. These findings suggest that lateral placenta, routinely assessed by ultrasound in the mid‐trimester, could serve as an additional perinatal risk indicator to guide closer surveillance. More studies that adjust for confounders are, however, needed to further clarify and strengthen the evidence base of this independent association.

## Author Contributions

J.A. and J.N. conceptualized the study, conducted searches, extracted data, and conducted risk of bias assessment. J.B., K.A.‐B., and K.B. contributed to the conceptualization of this study. J.A. and T.A.‐B. conducted data analysis. All authors contributed to the interpretation of findings and writing of the manuscript. The final version of the manuscript was reviewed and approved by all authors.

## Funding

This study received support from the University of Cape Coast Directorate of Research Innovation and Consultancy (UCC‐DRIC) research grant for evidence synthesis (DRIC‐RSG‐EVS‐24‐005).

## Ethics Statement

The authors have nothing to report.

## Conflicts of Interest

The authors declare no conflicts of interest.

## Supporting information


**Table S1:** Characteristics of included studies in systematic review and meta‐analysis.
**Table S2:** Excluded full‐text articles with reasons.
**Table S3:** Quality Assessment of included studies by the Newcastle–Ottawa Scale criteria.
**Table S4:** Studies reporting adjusted odds ratios (OR) after controlling for confounders.
**Figure S1:** Funnel plot for studies that reported on the association between lateral placenta and preeclampsia.
**Figure S2:** Funnel plot for studies that reported on the association between lateral placenta and small‐for‐gestational age.
**Figure S3:** Funnel plot for studies that reported on the association between lateral placenta and preterm birth < 37 weeks.
**Figure S4:** Subgroup analysis by study design for studies that reported on the association between lateral placenta and small‐for‐gestational age.
**Figure S5:** Meta‐analysis of the association between lateral placenta and caesarean section due to non‐reassuring foetal status.
**Figure S6:** Meta‐analysis of the association between lateral placenta and post‐partum haemorrhage.
**Figure S7:** Meta‐analysis of the association between lateral placenta and 5‐min Apgar score < 7.
**Figure S8:** Meta‐analysis of the association between lateral placenta and NICU admission.
**Figure S9:** Meta‐analysis of the association between lateral placenta and stillbirth.
**Figure S10:** Sensitivity analysis for studies that reported on the association between lateral placenta and preeclampsia.
**Figure S11:** Sensitivity analysis for studies that reported on the association between lateral placenta and small‐for‐gestational age.
**Figure S12:** Sensitivity analysis for studies that reported on the association between lateral placenta and preterm birth < 34 weeks.
**Figure S13:** Sensitivity analysis for studies that reported on the association between lateral placenta and preterm birth < 37 weeks.
**Figure S14:** Sensitivity analysis for studies that reported on the association between lateral placenta and retained placenta.
**Figure S15:** Sensitivity analysis for studies that reported on the association between lateral placenta and non‐vertex foetal presentation.
**Figure S16:** Secondary meta‐analysis of the association between lateral placenta and SGA by adjustment of confounders.
**Figure S17:** Secondary meta‐analysis of the association between lateral placenta and preterm < 34 weeks by adjustment of confounders.
**Figure S18:** Secondary meta‐analysis of the association between lateral placenta and preterm < 37 weeks by adjustment of confounders.
**Figure S19:** Secondary meta‐analysis of the association between lateral placenta and retained placenta by adjustment of confounders.
**Figure S20:** Secondary meta‐analysis of the association between lateral placenta and preeclampsia by adjustment of confounders.

## Data Availability

Data sharing not applicable to this article as no datasets were generated or analysed during the current study.
