# Supplementary material for: Association Between Lateral Placenta and Adverse Maternal and Perinatal Outcomes: A Systematic Review and Meta‐Analysis
Source: BJOG. 2026 Jun 7;133(12):2081–91. doi: 10.1111/1471-0528.70278 (PMC13659432; doi:10.1111/1471-0528.70278)
Supplement: Supplementary file 1 [file BJO-133-2081-s001.docx]

**Association between lateral placenta and adverse maternal and perinatal outcomes: A systematic review and meta-analysis.**

Joseph Arkorful^1,2^, Juliana Nyamson^2^, Theophilus Adu-Bredu^5^, Kwame Adu-Bonsaffoh^4^, Joyce Browne^3^, Albert D. Piersson^6^, Kitty Bloemenkamp^1^

**SUPPORTING INFORMATION**

**Appendix S1_Literature Search Strategy**

***All searches conducted across the Databases from inception to 25^th^ August, 2025***

| **Database (platform) with Search strategy** | **Number of records found** |
| --- | --- |
| **MEDLINE (via PUBMED)**  (Placentation[Title/Abstract] OR placenta* location[Title/Abstract] OR placenta* position[Title/Abstract] OR placenta* site[Title/Abstract] OR placenta* localization[Title/Abstract] OR placenta* localisation[Title/Abstract] OR central placenta*[Title/Abstract] OR anterior placenta*[Title/Abstract] OR posterior placenta*[Title/Abstract] OR lateral placenta*[Title/Abstract] OR “placentation”[MeSH])  AND  (Pregnancy outcome*[Title/Abstract] OR adverse pregnancy outcome*[Title/Abstract] OR neonatal outcome*[Title/Abstract] OR adverse neonatal outcome*[Title/Abstract] OR perinatal outcome*[Title/Abstract] OR adverse perinatal outcome*[Title/Abstract] OR obstetric outcome*[Title/Abstract] OR adverse obstetric outcome*[Title/Abstract] OR fetal outcome*[Title/Abstract] OR foetal outcome*[Title/Abstract] OR fetal death*[Title/Abstract] OR foetal death*[Title/Abstract] OR adverse fetal outcome*[Title/Abstract] OR adverse foetal outcome*[Title/Abstract] OR APGAR score[Title/Abstract] OR Neonatal intensive care[Title/Abstract] OR small for gestational age[Title/Abstract] OR intrauterine growth restriction[Title/Abstract] OR stillbirth[Title/Abstract] OR Fetal Growth Retard*[Title/Abstract] OR Feotal Growth Retard*[Title/Abstract] OR low birth weight*[Title/Abstract] OR Newborn Intensive Care Units*[Title/Abstract] OR NICU admission*[Title/Abstract] OR “pregnancy outcome”[MeSH] OR “APGAR score”[MeSH] OR “Intensive Care Units, Neonatal”[MeSH] OR “Fetal Growth Retardation” [MeSH] OR “Fetal Death”[MeSH] OR “infant, small for gestational age”[MeSH] OR “infant, low birth weight”[MeSH]) | 2, 127 |
| **Cochrane Library**  (‘Placentation’ OR ‘placenta* location’ OR ‘placenta* position’ OR ‘placenta* site’ OR ‘placenta* localization’ OR ‘placenta* localisation’ OR ‘central placenta*’ OR ‘anterior placenta*’ OR ‘posterior placenta*’ OR ‘lateral placenta*’)  **AND**  (‘Pregnancy outcome*’ OR ‘adverse pregnancy outcome*’ OR ‘neonatal outcome*’ OR ‘adverse neonatal outcome*’ OR ‘perinatal outcome*’ OR ‘adverse perinatal outcome*’ OR ‘obstetric outcome*’ OR ‘adverse obstetric outcome*’ OR ‘fetal outcome*’ OR ‘foetal outcome*’ OR ‘fetal death*’ OR ‘foetal death*’ OR ‘adverse fetal outcome*’ OR ‘adverse foetal outcome*’ OR ‘APGAR score’ OR ‘Neonatal intensive care’ OR ‘small for gestational age’ OR ‘intrauterine growth restriction’ OR ‘stillbirth’ OR ‘Fetal Growth Retard*’ OR ‘Feotal Growth Retard*’ OR ‘low birth weight*’ OR ‘Newborn Intensive Care Units’ OR ‘NICU admission*’) | 3, 801 |
| **SCOPUS (via ELSIVIER)**  **TITLE-ABS-KEY** (“Placentation” OR “placenta* location” OR “placenta* position” OR “placenta* site” OR “placenta* localization” OR “placenta* localisation” OR “central placenta*” OR “anterior placenta*” OR “posterior placenta*” OR “lateral placenta*”)  AND  **TITLE-ABS-KEY** (“Pregnancy outcome*” OR “adverse pregnancy outcome*” OR “neonatal outcome*” OR “adverse neonatal outcome*” OR “perinatal outcome*” OR “adverse perinatal outcome*” OR “obstetric outcome*” OR “adverse obstetric outcome*” OR “fetal outcome*” OR “foetal outcome*” OR “fetal death*” OR “foetal death*” OR “adverse fetal outcome*” OR “adverse foetal outcome*” OR “APGAR score” OR “Neonatal intensive care” OR “small for gestational age” OR “intrauterine growth restriction” OR “stillbirth” OR “Fetal Growth Retard*” OR “Feotal Growth Retard*” OR “low birth weight*” OR “Newborn Intensive Care Units” OR “NICU admission*”) | 2, 429 |
| **EMBASE (via ELSIVIER)**  (‘Placentation’:ti,ab,kw OR ‘placenta* location’:ti,ab,kw OR ‘placenta* position’:ti,ab,kw OR ‘placenta* site’:ti,ab,kw OR ‘placenta* localization’:ti,ab,kw OR ‘placenta* localisation’:ti,ab,kw OR ‘central placenta*’:ti,ab,kw OR ‘anterior placenta*’:ti,ab,kw OR ‘posterior placenta*’:ti,ab,kw OR ‘lateral placenta*’:ti,ab,kw OR ‘placentation’/exp)  AND  (‘Pregnancy outcome’:ti,ab,kw OR ‘adverse pregnancy outcome*’:ti,ab,kw OR ‘neonatal outcome*’:ti,ab,kw OR ‘adverse neonatal outcome*’:ti,ab,kw OR ‘perinatal outcome*’:ti,ab,kw OR ‘adverse perinatal outcome*’:ti,ab,kw OR ‘obstetric outcome*’:ti,ab,kw OR ‘adverse obstetric outcome*’:ti,ab,kw OR ‘fetal outcome*’:ti,ab,kw OR ‘foetal outcome*’:ti,ab,kw OR ‘fetal death*’:ti,ab,kw OR ‘foetal death*’:ti,ab,kw OR ‘adverse fetal outcome*’:ti,ab,kw OR ‘adverse foetal outcome*’:ti,ab,kw OR ‘APGAR score’:ti,ab,kw OR ‘Neonatal intensive care’:ti,ab,kw OR ‘small for gestational age’:ti,ab,kw OR ‘intrauterine growth restriction’:ti,ab,kw OR ‘stillbirth’:ti,ab,kw OR ‘Fetal Growth Retard*’:ti,ab,kw OR ‘Feotal Growth Retard*’:ti,ab,kw OR ‘low birth weight*’:ti,ab,kw OR ‘Newborn Intensive Care Units’:ti,ab,kw OR ‘NICU admission*’:ti,ab,kw OR ‘pregnancy outcome*’/exp OR ‘APGAR score’/exp OR ‘intensive care unit, newborn’/exp OR ‘intrauterine growth retardation’/exp OR ‘fetal; death’/exp OR ‘small for gestational age’/exp OR ‘low birth weight’/exp) | 4,760 |

**Table S1. Characteristics of Included Studies in Systematic review & Meta-analysis**

| **STUDY** | **COUNTRY OF ORIGIN** | **STUDY DESIGN** | **STUDY SAMPLE SIZE** | **GESTATIONAL AGE (in weeks)** | **PLACENTAL LOCATION INVESTIGATED** | **OUTCOMES INVESTIGATED** |
| --- | --- | --- | --- | --- | --- | --- |
| Fung (2011)^4^ | China | Retrospective cohort | 16236 | 14 – 24 | Central  Fundal  Lateral | - Preeclampsia  - SGA  - 5th min Apgar score < 7  - NICU admission  - Still birth  - Neonatal death  - Preterm birth  - Retained placenta  - Non-vertex presentation  - PPH  - CS- fetal compromise  - Fetal distress  - Low birth weight |
| Gizzo (2015)^29^ | Italy | Prospective cohort | 1056 | 29 – 31 | Anterior  Posterior  Fundal  Lateral | - Preterm birth |
| Seckin (2015)^10^ | Turkey | Retrospective cohort | 1052 | 18 – 24 | Central  Lateral | - 5th min Apgar score < 7  - SGA  - Preterm birth  - NICU admission  - Stillbirth  - Preeclampsia  - PPH  - CS- fetal compromise |
| Porto (2020)^2^ | Canada | Retrospective cohort | 1203 | ˃28 | Central  Lateral | - SGA  - 5th min Apgar score < 7  - NICU admission  - Preterm birth  - Preeclampsia |
| Doctory(2023)^11^ | Israel | Retrospective cohort | 12306 | 2^nd^ trimester | Central  Lateral | -SGA  - Preterm birth  - Preeclampsia |
| Adekanmi (2022)^3^ | Nigeria | Prospective cohort | 150 | 24 – 36 | Anterior  Posterior  Fundal  Lateral | - Preterm birth  - SGA  - Fetal distress  - Preeclampsia |
| Granfors (2019)^1^ | Sweden | Retrospective cohort | 74087 | 16 – 22 | Anterior  Posterior  Fundal  Lateral | - Preterm birth  - SGA  - 5th min Apgar score < 7 |
| Granfors (2020)^5^ | Sweden | Retrospective cohort | 3915 | 16 – 22 | Anterior  Posterior  Fundal  Lateral | - Retained placenta requiring manual removal |
| Magann (2007)^13^ | USA & Australia | Prospective cohort | 3336 | 14 – 22 | Fundal  Lateral | - 5th min Apgar score < 7  - Preterm birth  - SGA  - Stillbirth  - Non-vertex presentation  - PPH |
| Liberati (1997)^30^ | Italy | Retrospective cohort | 732 | 22 – 24 | Lateral  Central | - SGA |
| Shah (2021)^8^ | India | Prospective cohort | 397 | 18 – 24 | Lateral  Central | - SGA  - NICU admission |
| Salama-Bello (2019)^31^ | USA | Retrospective cohort | 464 | 18 – 24 | Lateral  Central | - SGA |
| Schreiber (2025)^15^ | Israel | Retrospective cohort | 23808 | ≥ 24 | Lateral  Anterior  Posterior  Fundal | - SGA  -Retained placenta requiring manual removal  - NICU admission  - 5th min Apgar score < 7  - Preterm birth  - Respiratory distress |
| Dagklis (2020)^32^ | Greece | Retrospective cohort | 5506 | 20 – 24 | Lateral  Central | - SGA  - Preeclampsia  - Preterm birth |
| Antsaklis (2000)^33^ | Greece | Prospective cohort | 654 | 20 – 32 | Lateral  Central | - Preeclampsia |
| Kakkar (2013)^34^ | India | Prospective cohort | 150 | 18 – 24 | Lateral  Central | - Preeclampsia |
| Lurie (2003)^36^ | Israel | Retrospective cohort | 200 | 3^rd^ trimester | Anterior  Posterior  Fundal  Lateral | - Retained placenta requiring manual removal |
| Meyer (2020)^35^ | Israel | Retrospective cohort | 16866 | 3^rd^ trimester | Lateral  Central | - Retained placenta requiring manual removal |
| Kalanithi (2007)^9^ | USA | Case-Control | 272 | 16 – 20 | Anterior  Posterior  Fundal  Lateral | - SGA |
| Alikhani (2024)^14^ | Iran | Case-Control | 206 | 14 – 26 | Lateral  Anterior  Posterior | - Preeclampsia |
| Contro (2010)^12^ | Italy | Case-Control | 131 | 20 – 24 | Lateral  Central | - Preeclampsia  - CS- fetal compromise  - Low birth weight |

*Abbreviations:*

SGA: small-for-gestational age; NICU: neonatal intensive care unit, CS: cesarean section, PPH: post-partum hemorrhage

**Table S2: Excluded full-text articles with reasons**

| **No** | **Article** | **Reasons for exclusion** |
| --- | --- | --- |
| 1 | Devarajan, K., Kives, S. & Ray, J. G. Placental location and newborn weight. *J Obstet Gynaecol Can* **34**, 325–329 (2012). | Unclear categorization of placental location |
| 2 | Zia, S. Placental location and pregnancy outcome. *J Turk Ger Gynecol Assoc* **14**, 190–193 (2013). | Did not assess lateral placenta within the study cohort |
| 3 | Torricelli, M. *et al.* Anterior placental location influences onset and progress of labor and postpartum outcome. *Placenta* **36**, 463–466 (2015) | Did not assess lateral placenta within the study cohort |
| 4 | Babayan, A., Hellmann, R., Struk, I. & Roberts, M.-C. The Effects of Placental Location on Maternal and Neonatal Outcomes: A Retrospective Cohort Study. *Journal of Diagnostic Medical Sonography* **40**, 361–366 (2024) | Did not assess lateral placenta within the study cohort |
| 5 | Kofinas, A. D., Penry, M., Swain, M. & Hatjis, C. G. Effect of placental laterality on uterine artery resistance and development of preeclampsia and intrauterine growth retardation. *American Journal of Obstetrics & Gynecology* **161**, 1536–1539 (1989). | Ambiguity in reporting placental location, i.e. unclear if low-lying placenta and placenta previa were included in cohort |
| 6 | Ogueh, O., Morin, L., Usher, R. h. & Benjamin, A. Obstetric implications of low-lying placentas diagnosed in the second trimester. *International Journal of Gynecology & Obstetrics* **83**, 11–17 (2003). | Investigated adverse fetal and neonatal outcomes in women with low-lying placenta or previa only |
| 7 | Kim, O., Hong, S., Park, I. Y. & Ko, H. S. Association between placental location and cord insertion site with pre-eclampsia: a retrospective cohort study. *J Matern Fetal Neonatal Med* **37**, 2306189 (2024). | Did not assess lateral placenta within the study cohort |
| 8 | Osmundson, S. S., Wong, A. E. & Gerber, S. E. Second-trimester placental location and postpartum hemorrhage. *J Ultrasound Med* **32**, 631–636 (2013). | Investigated adverse maternal outcomes in women with low-lying placenta |

**Table S3. Quality Assessment of included studies by the Newcastle-Ottawa Scale criteria**

Cohort studies

|  |  | **Selection** | | | | **Comparability** | | **Outcome** | |  |
| --- | --- | --- | --- | --- | --- | --- | --- | --- | --- | --- |
| **Study** | **Study Design** | Exposed representation | Selection of the non-exposed | Ascertainment of exposure | Outcome of interest was not present at start of study | Comparability of cohorts  (*Controlling for important confounders*) | Assessment of outcome | Was follow-up long enough for outcomes to occur | Adequacy of follow-up of cohorts | **Total score*** |
| Fung (2011)^4^ | Retrospective cohort | * | * | * | * | ** | * | * | * | **9** |
| Gizzo (2015)^29^ | Prospective cohort | * | * | * | * | - | * | * | * | **7** |
| Seckin (2015)^10^ | Retrospective cohort | * | * | * | * | - | * | * | * | **7** |
| Porto (2020)^2^ | Retrospective cohort | * | * | * | * | ** | * | * | * | **9** |
| Doctory(2023)^11^ | Retrospective cohort | * | * | * | * | ** | * | * | * | **9** |
| Adekanmi (2022)^3^ | Prospective cohort | * | * | * | * | - | * | * | * | **7** |
| Granfors (2019)^1^ | Retrospective cohort | * | * | * | * | ** | * | * | * | **9** |
| Granfors (2020)^5^ | Retrospective cohort | * | * | * | * | ** | * | * | * | **9** |
| Magann (2007)^13^ | Prospective cohort | * | * | * | * | ** | * | * | * | **9** |
| Liberati (1997)^30^ | Retrospective cohort | * | - | * | * | - | * | * | * | **6** |
| Shah (2021)^8^ | Prospective cohort | * | * | * | * | - | * | * | * | **7** |
| Salama-Bello (2019)^31^ | Retrospective cohort | * | * | * | * | - | * | * | * | **7** |
| Schreiber (2025)^15^ | Retrospective cohort | * | * | * | * | * | * | * | * | **8** |
| Dagklis (2020)^32^ | Retrospective cohort | * | * | * | * | - | * | * | * | **7** |
| Antsaklis (2000)^33^ | Prospective cohort | * | * | * | * | - | * | * | * | **7** |
| Kakkar (2013)^34^ | Prospective cohort | * | * | * | * | - | * | * | * | **7** |
| Lurie (2003)^36^ | Retrospective cohort | * | * | * | * | - | * | * | * | **7** |
| Meyer (2020)^35^ | Retrospective cohort | * | * | * | * | - | * | * | * | **7** |

Case-Control studies

|  |  | **Selection** | | | | **Comparability** | | **Exposure** | | |  |
| --- | --- | --- | --- | --- | --- | --- | --- | --- | --- | --- | --- |
| **Study** | **Study Design** | Adequate definition of cases | Representativeness of cases | Selection of controls | Definition of controls | Control for important confounders | Control for additional confounders | Ascertainment of exposure | Same method of ascertainment for cases and controls | Non-response rate | **Total score** |
| Kalanithi (2007)^9^ | Case-control | * | * | * | * | * | * | * | * | **-** | **8** |
| Alikhani (2024)^14^ | Case-Control | * | * | * | * | - | - | * | * | **-** | **6** |
| Contro (2010)^12^ | Case-Control | * | * | * | * | - | - | * | * | **-** | **6** |

*Study Quality level: High (score >7 ); Moderate (score 5 – 7); Low (score < 5)

**Table S4. Studies reporting adjusted odds ratios (OR) after controlling for confounders**

| ***Small-for-gestational age (SGA)*** | | | |
| --- | --- | --- | --- |
| *Study* | *Study Country of origin* | *Confounders adjusted for* | *Adjusted OR(95% CI)* |
| Fung (2011)^4^ | China | Advanced maternal age,  ethnicity, nulliparity, maternal body mass index at booking and year of delivery, gestational diabetes mellitus, pre-existing hypertension, pre-existing diabetes mellitus, antepartum hemorrhage | 1.82 (1.48–2.23) |
| Granfors (2019)^1^ | Sweden | Advanced maternal age, maternal height, maternal country of birth, smoking in early pregnancy,  sex of the infant and use of in vitro fertilization. | 1.79 (1.40 - 2.27) |
| Kalanithi (2007)^9^ | USA | Age, race, chronic hypertension, hypertensive disorders of pregnancy | 3.80 (1.30 – 11.2) |
| ***Preterm birth <37weeks*** | | | |
| *Study* | *Study Country of origin* | *Confounders adjusted for* | *Adjusted OR(95% CI)* |
| Fung (2011)^4^ | China | advanced maternal age,  ethnicity, nulliparity, maternal body mass index at booking, year of delivery, preeclampsia/eclampsia, gestational diabetes mellitus, pre-existing hypertension, pre-existing diabetes mellitus, any antepartum hemorrhage | 1.46 (1.08 – 1.99) |
| Porto (2020)^2^ | Canada | parity, small-for-gestational age, hypertensive disorders of pregnancy | 2.99 (1.43 – 6.14) |
| Doctory(2023)^11^ | Israel | maternal age, parity, body mass index, ethnicity, smoking, and infant sex | 1.36 (1.11–1.66) |
| Granfors (2019)^1^ | Sweden | maternal age, maternal height, maternal country of birth, smoking in early pregnancy, sex of the infant and use of in vitro fertilization*.* | 1.62 (1.32 – 2.00) |
| ***Preterm birth <34weeks*** | | | |
| *Study* | *Study Country of origin* | *Confounders adjusted for* | *Adjusted OR(95% CI)* |
| Fung (2011)^4^ | China | advanced maternal age,  ethnicity, nulliparity, maternal body mass index at booking, year of delivery, preeclampsia/eclampsia, gestational diabetes mellitus, pre-existing hypertension, pre-existing diabetes mellitus, any antepartum hemorrhage, placenta previa. | 1.96 (1.16 – 3.32) |
| Porto (2020)^2^ | Canada | parity, small-for-gestational age, hypertensive disorders of pregnancy | 3.92 (1.01 – 15.24) |
| Granfors (2019)^1^ | Sweden | maternal age, maternal height, maternal country of birth, smoking in early pregnancy, sex of the infant and use of in vitro fertilization*.* | 2.13 (1.39 – 3.26) |
| ***Retained placenta requiring manual removal of placenta*** | | | |
| *Study* | *Study Country of origin* | *Confounders adjusted for* | *Adjusted OR(95% CI)* |
| Fung (2011)^4^ | China | advanced maternal age,  ethnicity, nulliparity, maternal body mass index at booking and year of delivery, history of previous abortion/miscarriage | 6.75 (4.40 – 10.33) |
| Granfors (2019)^1^ | Sweden | maternal age, height, smoking in early pregnancy, and country of birth, use of in vitro fertilization and sex of the infant. | 3.31 (2.64 – 4.16) |
| Granfors (2020)^5^ | Sweden | maternal age, height, smoking in early pregnancy, and country of birth, use of in vitro fertilization and sex of the infant | 4.03 (1.97 – 8.23) |
| **Preeclampsia** | | | |
| *Author(year)* | *Study Country of origin* | *Confounders adjusted for* | *Adjusted OR(95% CI)* |
| Fung (2011)^4^ | China | advanced maternal age,  ethnicity, nulliparity, maternal body mass index at booking and year of delivery pre-existing hypertension | 2.04 (1.28 – 3.25) |
| Granfors (2019)^1^ | Sweden | maternal age, maternal height, maternal country of birth, smoking in early pregnancy,  sex of the infant and use of in vitro fertilization | 1.32 (1.04 – 1.67) |

**Figure S1: Funnel plot for studies that reported on the association between lateral placenta and preeclampsia**

**Figure S2: Funnel plot for studies that reported on the association between lateral placenta and small-for-gestational age**

**Figure S3: Funnel plot for studies that reported on the association between lateral placenta and preterm birth <37weeks**

**Figure S4: Subgroup analysis by study design for studies that reported on the association between lateral placenta and small-for-gestational age**

**Figure S5: Meta-analysis of the association between lateral placenta and cesarean section due to non-reassuring fetal status**

**Figure S6: Meta-analysis of the association between lateral placenta and post-partum hemorrhage**

**Figure S7: Meta-analysis of the association between lateral placenta and 5-minute Apgar score <7**

**Figure S8: Meta-analysis of the association between lateral placenta and NICU admission**

**Figure S9: Meta-analysis of the association between lateral placenta and stillbirth**

**Figure S10: Sensitivity Analysis for studies that that reported on the association between lateral placenta and preeclampsia**

**Figure S11: Sensitivity Analysis for studies that that reported on the association between lateral placenta and small-for-gestational age**

**Figure S12: Sensitivity Analysis for studies that that reported on the association between lateral placenta and preterm birth <34weeks**

**Figure S13: Sensitivity Analysis for studies that that reported on the association between lateral placenta and preterm birth <37weeks**

**Figure S14: Sensitivity Analysis for studies that that reported on the association between lateral placenta and retained placenta**

**Figure S15: Sensitivity Analysis for studies that that reported on the association between lateral placenta and non-vertex fetal presentation**

**Figure S16: Secondary meta-analysis of the association between lateral placenta and SGA by adjustment of confounders**

**Figure S17: Secondary meta-analysis of the association between lateral placenta and preterm <34weeks by adjustment of confounders**

**Figure S18: Secondary meta-analysis of the association between lateral placenta and preterm <37weeks by adjustment of confounders**

**Figure S19: Secondary meta-analysis of the association between lateral placenta and retained placenta by adjustment of confounders**

**Figure S20: Secondary meta-analysis of the association between lateral placenta and preeclampsia by adjustment of confounders**
